# Scanning electron microscopy observations of the hedgehog stomach worm, *Physaloptera clausa* (Spirurida: Physalopteridae)

**DOI:** 10.1186/1756-3305-6-87

**Published:** 2013-04-08

**Authors:** Tahmine Gorgani, Soraya Naem, Amir Abbass Farshid, Domenico Otranto

**Affiliations:** 1 Department of Pathobiology, Faculty of Veterinary Medicine, Urmia University, PO BO Box 1177, Urmia, 57153, Iran; 2 Department of Veterinary Medicine University of Bari, Str. prov. per Casamassima km 3, Valenzano, Bari, 70010, Italy

**Keywords:** Hedgehog, Nematode, *Physaloptera clausa*, Morphology, Scanning electron microscopy

## Abstract

**Background:**

*Physaloptera clausa* (Spirurida: Physalopteridae) nematodes parasitize the stomach of the European hedgehog (*Erinaceus europaeus*) and cause weight loss, anorexia and gastric lesions. The present study provides the first morphological description of adult *P. clausa* from the stomachs of infected hedgehogs, using scanning electron microscopy (SEM).

**Methods:**

From June to October 2011, 10 *P. clausa* from European hedgehogs were fixed, dried, coated and subjected to SEM examination.

**Results:**

Males and females (22–30 mm and 28–47 mm, respectively) were stout, with the cuticle reflecting over the lips to form a large cephalic collarette and showing fine transverse striations in both sexes. The mouth was characterized by two large, simple triangular lateral pseudolabia, each armed with external and internal teeth. Inside the buccal cavity, a circle of internal small teeth can be observed. Around the mouth, four sub-median cephalic papillae and two large amphids were also observed. The anterior end of both male and female bore an excretory pore on the ventral side and a pair of lateral ciliated cervical papillae. In the female worm, the vulva was located in the middle and the eggs were characterized by smooth surfaces. The posterior end of the female worm was stumpy with two large phasmids in proximity to its extremity. The posterior end of the male had large lateral alae, joined together anteriorly across the ventral surface, with subequal and dissimilar spicules, as well as four pairs of stalked pre-cloacal papillae, three pairs of post-cloacal papillae, and two phasmids. Three sessile papillae occured anteriorly and four posteriorly to the cloaca.

**Conclusions:**

The present SEM study provides the first in-depth morphological characterization of adult *P. clausa*, and highlights similarities and differences with *P. bispiculata P. herthameyerae, Heliconema longissimum* and *Turgida turgida*.

## Background

Adult nematodes of the genus *Physaloptera* Rudolphi 1819, occur in the stomach and seldom in the small intestine of amphibians, reptiles, birds and a wide range of insectivorous and omnivorous mammals throughout the world [1-6]. The first record of *Physaloptera* from a marsupial was that of Ortlepp [1], who described a species from a long-nosed bandicoot *Perameles nasuta* Geoffroy, which died at the London Zoo. Ortlepp did not name the species, because the asymmetry of the ventral caudal papillae on the tail of the male was abnormal [1,7]. In 1999, Norman and Beveridge redescribed the *Physaloptera* species (*P. peramelis*, *P. thalacomys,* and *Physaloptera* sp. from both *Perameles nasuta* and *Isoodon macrourus*) in bandicoots (Marsupialia: Perameloidea) in Australia [7]. Adult females are ovoviviparous, and the life cycle involves insects including beetles, cockroaches, and crickets as intermediate hosts and reptiles as paratenic hosts [8-13]. The parasite is usually firmly attached to the mucosa of the definitive host, from where they feed on blood. It has been reported [14] that the parasites may occasionally change their site of attachment, therefore causing the formation of numerous small oedematous ulcers, and consequently bleeding, inflammation and increased mucus production. Heavy infections lead to anaemia, weakness, anorexia, diarrhea, cachexia, and weight loss. Diagnosis often relies on the observation of clinical signs and parasite eggs in the faeces of the vertebrate host [14].

*Physaloptera clausa* causes severe gastric lesions in the European hedgehog (*Erinaceus europaeus*), and has been reported from Israel, Italy, Germany, Turkey, and Poland [15-19]. To date, there is a paucity of information of the morphology of species within the families Physalopteridae and Spiruridae [6,20-30]. The present study addresses this gap in knowledge by reporting the first SEM description of adult *P. clausa* from the stomachs of infected hedgehogs.

## Methods

European hedgehogs are spiny mammals that have become popular as exotic pets in this area. These animals play a role in the transmission of a wide variety of viruses, bacteria, fungi and parasites, some of which have zoonotic importance [31]. For example, it is suggested that hedgehogs may be host of *Trichinella* spp in some parts of the world [32]. These animals are infected with gastrointestinal nematodes. One of the parasites in hedgehogs is *Physaloptera clausa* a common species in the family of Physalopteridae. Adult females are ovoviviparous and arthropod species act as intermediate hosts. In the infected infested animals, large numbers of parasites can lead to weakness, cachexia, anaemia, light diarrhea, and weight loss. In addition, *P. clausa* can serve as a vector of leptospirosis providing a contamination source for animals and humans [15]. Hence, both hedgehog and *P. clausa* might be important to be studied.

From June to October 2011, twelve carcasses (some were found dead in farms and some were killed by motor vehicles on the road) of European hedgehogs (6 females and 6 males) were referred to the Department of Pathobiology, at the Faculty of Veterinary Medicine, Urmia University, Iran. Parasitological and histopathological examinations were carried out specifically for the collection of stomach nematodes. At necropsy, congestion of the attaching site of nematodes and oedema, erosion and a thickened stomach wall were observed. A total of 10 worms (5 females and 5 males) were selected for the SEM examination. The worms were washed in 2% sodium cacodylate buffer (pH 7.2), and then cleared with lactophenol solution (25% glycerine, 25% lactic acid, 25% phenol, 25% distilled water). Specimens were identified as *P. clausa* based on morphological features such as cephalic arrangement; distance of vulva from anterior end of the female; number, size, and position of male caudal papillae; cuticular ornamentation of the ventral part of the mail tail; shape and size of spicules [2,3,33-37]. The worms were subsequently placed in 4% glutaraldehyde and the examined at the Department of Pathology and Molecular Medicine, McMaster University, Canada. The nematodes were put into fresh 4% glutaraldehyde for 2 h, post fixed in 1% osmium tetraoxide in cacodylate buffer (pH 7.2) at 4°C for 1 h and dehydrated through a series of increasing ethanol concentrations (50, 75, 96 and 100%). Worms were then dried with liquid CO_2_ in a critical point drier apparatus (Critical PointDrier, Model no. 2800, Ladd Research Industries, Burlington,VT, USA). Each worm was placed on a numbered aluminium stub, which was coated with gold in an ion coating sputter device (Sputter Coater, Model no. E5100, Polaron Instruments, USA) and examined with a JEOL JSM-840 Scanning Electron Microscope operated at 20 kV. Photographs were taken with a digital acquisition system for analog SEMs (GW Electronics, Norcross, GA, USA).

## Results

**Figure 1:**
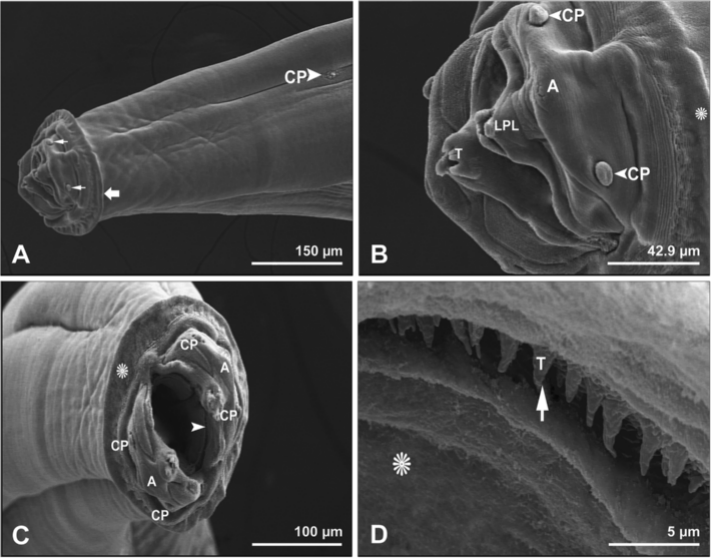
**Scanning electron micrographs showing anterior end of female *Physaloptera clausa*. A**, Cephalic papillae (thin arrows), cephalic collarette (thick arrow), and ciliated cervical papilla (CP). Bar= 150 μm. **B**, Triangular lateral pseudolabia (LPL), external teeth (T), one of two amphids (A), two of four cephalic papillae (CP), and cephalic collarette (star). Bar= 42.9 μm. **C**, Two amphids (A), four cephalic papillae (CP), cephalic collarette (star), and a circle of internal small teeth inside the mouth (arrowhead). Bar= 100 μm. **D**, Higher magnification of inside of the mouth (star), and internal small teeth (T). Bar= 5 μm.

All worms removed from the stomach of the examined hedgehogs were identified as *P. clausa*. The worms were white in colour, stout and the cuticle was reflected over the lips to form a large cephalic collarette (Figures 1A-C). Around the buccal opening, four cephalic papillae and two large amphids were observed (Figures 1B,C). The buccal cavity, surrounded by a thick wall, was cylindrical and characterized by two large, simple triangular lateral pseudolabia, each armed with external (Figure 1B) and internal (Figures 1C,D) teeth.

**Figure 2:**
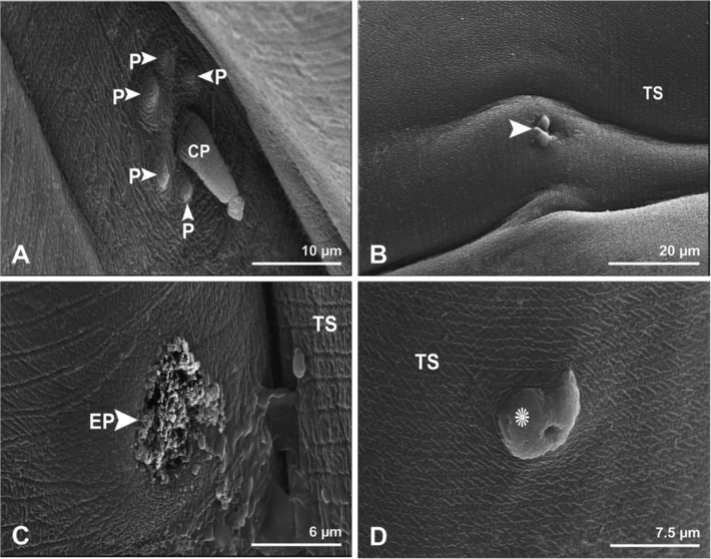
**Scanning electron micrographs showing anterior end of female *Physaloptera clausa*. A**, Higher magnification of ciliated cervical papilla (CP), and a cluster of small papillae (P). Bar= 10 μm. **B**, Higher magnification of an abnormal ciliated cervical papilla (arrowhead), and cuticular transverse striations (TS). Bar= 20 μm. **C**, Higher magnification of the excretory pore (EP), and cuticular transverse striations (TS). Bar= 20 μm. **D**, Higher magnification of a single papilla (star), and cuticular transverse striations (TS). Bar= 7.5 μm.

An excretory pore and a pair of lateral ciliated cervical papillae with a cluster of small papillae were observed on the ventral surface of the anterior end of both male and female (Figures 2A,C). The cuticle was characterized by fine transverse striations in both sexes (Figures 2B-D). In one female worm, an abnormal ciliated cervical papilla was observed (Figure 2B). All female worms bore a single papilla 589 μm from the anterior end (Figure 2D).

**Figure 3:**
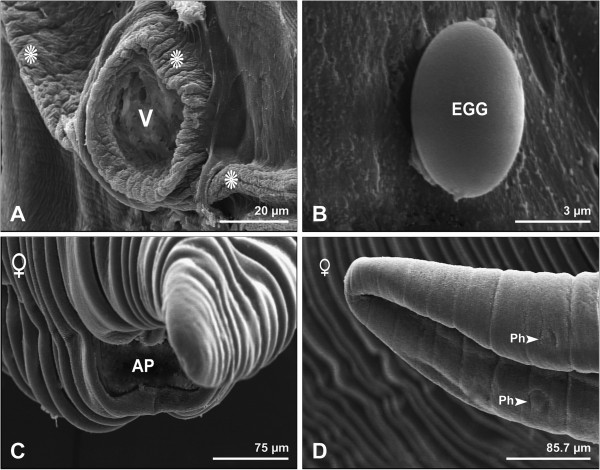
**Scanning electron micrographs showing female *Physaloptera clausa*. A**, Vulva (V), and the cuticle around the vulva (stars). Bar= 20 μm. **B**, Egg (EGG). Bar= 3 μm. **C,** Anal pore (AP). Bar= 75 μm. **D**, Higher magnification of phasmids (Ph). Bar= 85.7 μm.

The females were 28–47 mm long and 1.3-1.6 mm wide. The female vulva was located in front of the middle of the body. The pattern of the cuticle around the vulva differed from the cuticular transverse striations (Figure 3A). The eggs were oval, with smooth surfaces and measured 38×52 μm (Figure 3B). The anal pore was located at the posterior end (Figure 3C). At the blunted rounded posterior end, two subterminal large phasmids were observed, approximately 222 μm from the tail end (Figure 3D).

**Figure 4:**
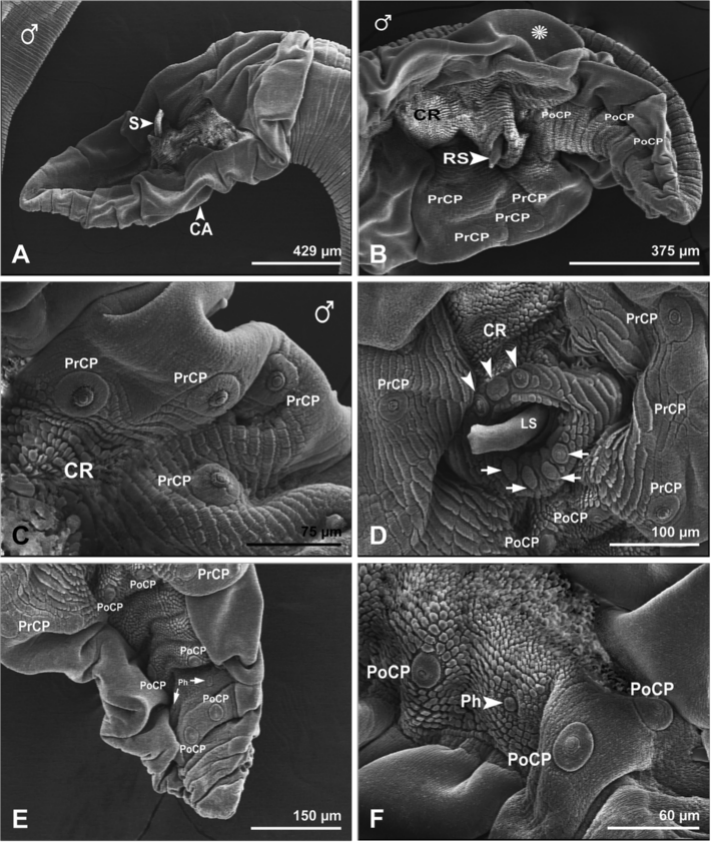
**Scanning electron micrographs showing posterior end of male *Physaloptera clausa*. A**, caudal alae (CA), and spicule (S). Bar= 429 μm. **B**, Caudal alae (star), four stalked precloacal papillae (PrCP), three postoacal papillae (PoCP), and cuticular ridges (CR). Bar= 375 μm. **C**, Higher magnification of four of stalked precloacal papillae (PrCP), and cuticular ridges (CR). Bar= 75 μm. **D**, Stalked precloacal papillae (PrCP), postcloacal papillae (PoCP), three (arrowhead) and four (arrows) sessile papillae directly to anterior and posterior to cloaca, respectively,left spicule (LS), and cuticular ridges (CR). Bar= 100 μm. **E**, Stalked precloacal papillae (PrCP), postcloacal papillae (PoCP), and phasmids (Ph). Bar= 150 μm**. F**, Higher magnification of postcloacal papillae (PoCP), and phasmid (Ph). Bar= 60 μm.

The males were 22–30 mm long and 0.82-1.31 mm wide. The male tail bore large lateral alae, meeting ventrally in front of the cloaca; the spicules were subequal and dissimilar (Figure 4A,B,D). While the right spicule was characterized by a sharp tip (Figure 4B), the left spicule had a thick extremity (Figures 4A,D). Anteriorly and posteriorly to the cloaca, three and four sessile papillae, respectively, were observed (Figure 4D). The posterior extremity of the male was characterized by the presence of four pairs of stalked pre-cloacal papillae (Figure 4B-D), three pairs of post-cloacal papillae (Figure 4D-F) and two phasmids (Figure 4E,F).

## Discussion

The present SEM study provides morphological characterizations of both male and female *P. clausa*, for the first time. The cephalic region, female’s vulva, egg, anal pore and phasmids were shown. In the male worm, morphological details of the posterior end including pre-cloacal and post-cloacal papillae, sessile papillae, spicules, phasmids, and cuticular ridges were described. Also, similarities and differences with *P. bispiculata, P. herthameyerae, Heliconema longissimum* and *Turgida turgid* were discussed.

The cephalic structures of *P. clausa* were similar to those of *P. bispiculata*[22]. The mouth of *P. clausa* has two large, simple triangular lateral pseudolabia, each armed with external teeth, with a circle of internal small teeth inside the buccal cavity; in contrast, the cephalic end of *P. bispiculata* is dome-shaped, composed of two semicircular lips laterally surrounding the oral opening. The lips are flattened on the inner face and undivided. Each lip carries a pair of comma-shaped cephalic papillae symmetrically situated on the surface of a hemi dome [22]. Similarly to *P. bispiculata*, in addition to the large tripartite tooth, several smaller ones are present along the inner border of each lip of *P. clausa*[22]. In another SEM study of *P. herthameyerae* n. sp., the cephalic end was composed of two semicircular pseudolips, laterally surrounding the oral opening [27]. Each lip bore a pair of cephalic papillae, three porous-like circumscribed regions, with a cuticle pattern differing from the surface of the anterior portion. The internal margins of the lips was characterized by a pair of cuticular folds, with a tripartite tooth projected between them, each one bearing a pore on the inner surface and a fourth external tooth [27]. Similarly, the oral aperture of another Physalopterid nematode, *Heliconema longissimum,* is surrounded by two lateral pseudolabia. Each pseudolabium bears two large submedian cephalic papillae and oval latero-terminal depressions with a small lateral amphid situated at the base of each pseudolabium. Also, the inner surface of each pseudolabium is characterized by a triangular tooth and simple flat tooth at each dorsoventral extremity [26]. The numerous denticles on the inner side of the buccal cavity of *P. clausa* are similar to those observed in *Turgida turgida*, a parasitic nematode of the Virginia opossum *Didelphis virginiana*, first reported by Matey *et al*. [23]. The function of these denticles is yet unclear, albeit a function for attachment to the stomach wall has been hypothesized [38]. In the present study, two lateral amphids were observed. Nematode amphids exist in a variety of forms and sizes, some probably purely chemosensory and some photoreceptive, with an associated gland [39]. The functional type of the amphids of *P. clausa* is still unknown. Also, a pair of lateral ciliated cervical papillae with a cluster of small papillae was observed at the anterior end of both male and female *P. clausa.* The cervical papillae are often very small and inconspicuous, which has led to the erroneous conclusion that they are absent in some species. The larger, conspicuous cervical papillae vary in shape, from thin, needle-like appendages to complex structures with denticulate posterior borders. The position, size and shape of the cervical papillae are used as taxonomic characters. Given their structure and position, it is likely that they act as mechano-receptors [40], essential for their passage through a small space [41].

The vulva of *P. clausa* was located in front of the middle of the body. The vulvar aperture of *P. bispiculata* is located in the anterior region, posterior to the esophagus [22], while the vulvar opening of *P. herthameyerae* was 5.71-10.1 mm from the anterior end [27]. In *H. longissimum*, the vulva is situated at 39-66% of body length from the apical extremity, while it is located 1.14-1.36 mm from the posterior end of the body of *P. obtusus*[24,26]. The presence of eggs in the male reproductive system of *P. bispiculata* was described by Oliveira-Menezes *et al.* in 2010 [42]. To the best of our knowledge, this is the first report of a male physalopterid nematode harbouring eggs in the cloacal region, ejaculatory duct or intestine. Several eggs were observed glued by cement to the cloacal aperture, using scanning electron microscopy [42]. Light microscopy revealed that some males had an uncountable number of embryonated eggs in the ejaculatory duct, cloaca and also in the posterior part of the intestine [42]. The probable explanation is that the eggs developing in the female uterus are pumped by the female or sucked by the male to the cloacal opening and from this point to the intestine and ejaculatory duct. The male worm may be unable to expel the eggs and a large number were found in these organs. In addition, this could be an important adaptation for the parasite, for example the males expelled by the host can carry a large number of eggs and spread them to intermediate hosts when ingested by these hosts [42]. The vulva of *T. turgida* is located in the anterior end of the body, and like in *P. clausa*, the conical tail bore an anal opening, with phasmids located posteriorly [23]. Phasmids are involved in the evaluation of the intensity of a given stimulus and help the worm to maintain itself and stabilize in a suitable environment [41].

The number of caudal papillae of male *P. clausa* was different from that of male *P. bispiculata, P. obtusus* and *H. longissimum*[22,24,26], but similar to that of *P. herthameyerae*[27]. In this study, the male tail bore large lateral alae meeting ventrally in front of the cloaca, while three and four sessile papillae were observed anteriorly and posteriorly to the cloaca, respectively. Also, four pairs of stalked pre-cloacal papillae, three pairs of post-cloacal papillae, and two phasmids were present. The posterior end of male *P. herthameyerae* was ventrally bent and the cuticle appeared loose, forming lateral caudal alae. The transverse striations of the body terminated in correspondence to the alae and the ventral region bore rows of cuticular bead-like structures, forming small ridges flanking the cloaca and the three precloacal papillae. The central area posterior to the second pair of post-cloacal papillae was smooth towards the tail tip. The 21 papillae were button-like. Three were situated anterior of the cloacal opening, while of the five pairs of post-ventral papillae, two pairs were located on a protuberance posterior to the cloaca and three pairs between the cloaca and the end of the tail. Four pedunculated papillae were observed each side of the cloaca, together with two phasmids [27]. The posterior end of male *H. longissimum* was spirally coiled, supported by four twin pairs of pedunculate pre-anal papillae and six single pairs of post-anal papillae, of which each of the first four pairs were large, while those of the last two pairs were small and sessile; an additional pair of smaller post-anal sessile papillae were situated ventrally at the level of the first post-anal pair. Each caudal papilla was surrounded by a ring consisting of numerous cuticular, papilla-like protuberances [26]. In the present study, the spicules of *P. clausa* were subequal and dissimilar; the right spicule was characterized by a sharp tip, whereas the left spicule by a thickened end. Spicules of *H. longissimum* were unequal and dissimilar, the left spicule with a sharply pointed distal tip and the distal half alate except for a conical tip; the right spicule was broader, boat-shaped, and tapered toward the distal tip [26]. The tail of the male *P. bispiculata* was ventrally bent and the cuticle appeared loose in the region anterior to the cloaca, with two folds. Between these structures and the cloaca there was a deep cuticular transverse fold [22]. The 21 button-like caudal papillae were also observed at the posterior end of male *P. bispiculata*. The phasmids, which were well-delimited round structures with pores and bumps on their surface, were located between the fourth and fifth pairs of posteroventral papillae [22]. In 2001, Matey *et al.* speculated that the general arrangement of male caudal papillae in *T. turgida* is the same as in *P. praeputialis*, *P. rara*, and *P. bispiculata*[23]. The unique feature of male *T. turgida* is the presence of the 22^nd^ broad caudal papilla, which had already been observed by Gray and Anderson in 1982 [11], who considered it a sessile papilla. The broad papilla may represent a distinct type of sensory structure [23]. Like *P. rara* and *P. bispiculata*, and in contrast to *P. praeputialis*, *T. turgida* has paired phasmids [11,22,43]. In the present study, the pattern of the cuticle of male *P. clausa* at the cloacal region was different and covered with small cuticular ridges. The ornamentation of the ventral surface of the male tail of *T. turgida,* like *P. bispiculata,* includes three different patterns that present some similarities and differences with the cuticular features of the male tail in other physalopterids [22,23].

## Conclusions

The results of previous and present studies indicate that morphological features; such as cephalic arrangement; number of uteri; distance of vulva from anterior end of the female; number, size, and position of male caudal papillae; cuticular ornamentation of the ventral part of the male tail; shape and size of spicules are important features which should be considered in the classification of physalopterids. The information provided herein will contribute towards the elucidation of the biology of this little known parasite. Although, the function of external and internal teeth on triangular lateral pseudolabiae, the numerous denticles on the inner side of the buccal cavity of *P. clausa*, is still unknown, but they may have an important role in the nematode’s feeding during its attachment to the stomach wall, and cause serious tissue damage as were observed at necropsy. Meanwhile, structures such as amphids, phasmids, ciliated cervical papillae with a cluster of small papillae, caudal papillae, sessile papillae, and spicules described herein using SEM, deserve further investgation using transmission electron miscroscope (TEM) directed toward determining their functions and to (?) provide more data on the biology of the parasite.

## Competing interests

The authors declare that they have no competing interests.

## Authors’ contributions

TG: examined the hedgehogs, removed the worms and performed parasitological process using light microscope. SN: designed the project, transferred the samples to McMaster University, Canada, and prepared the samples for SEM study, edited SEM micrographs, and drafted the manuscript. AAF: participated in designing the study and planning the work. DO: participated in designing study, commented on SEM micrographs, drafted the manuscript, and revised the paper. All authors reviewed and approved the manuscript.
